# An Efficient Alert Aggregation Method Based on Conditional Rough Entropy and Knowledge Granularity

**DOI:** 10.3390/e22030324

**Published:** 2020-03-12

**Authors:** Jiaxuan Sun, Lize Gu, Kaiyuan Chen

**Affiliations:** Institute of Cyberspace Security, Beijing University of Posts and Telecommunications, Beijing 100876, China

**Keywords:** conditional rough entropy, knowledge granularity, attribute similarity, alert aggregation

## Abstract

With the emergence of network security issues, various security devices that generate a large number of logs and alerts are widely used. This paper proposes an alert aggregation scheme that is based on conditional rough entropy and knowledge granularity to solve the problem of repetitive and redundant alert information in network security devices. Firstly, we use conditional rough entropy and knowledge granularity to determine the attribute weights. This method can determine the different important attributes and their weights for different types of attacks. We can calculate the similarity value of two alerts by weighting based on the results of attribute weighting. Subsequently, the sliding time window method is used to aggregate the alerts whose similarity value is larger than a threshold, which is set to reduce the redundant alerts. Finally, the proposed scheme is applied to the CIC-IDS 2018 dataset and the DARPA 98 dataset. The experimental results show that this method can effectively reduce the redundant alerts and improve the efficiency of data processing, thus providing accurate and concise data for the next stage of alert fusion and analysis.

## 1. Introduction

With the continuous development of computer network technology, people are becoming increasingly dependent on the convenience of the Internet. At the same time, the diversity, openness, connectivity, and other characteristics of the network are leading to the diversification and complexity of security threats. A large number of different network security technologies have been used widely, such as the intrusion detection system (IDS), firewall, vulnerability scanner, etc., in order to protect devices on the Internet from illegal intrusion. How to deal with the large amount of alert data that are generated by these security devices, especially the intrusion detection system, and discover security events, has become an important research topic in practical applications.

Intrusion detection can be divided into anomaly detection and misuse detection, according to different detection techniques. Anomaly detection assumes that the activity of an intruder is different from that of a normal user. The system collects data from normal users and obtains a normal behavior pattern through analysis and processing. During the detection process, the data to be detected are compared with the normal behavior model. When the model cannot be matched, the activity is considered to be an intrusion behavior [1]. In the misuse detection method, the abnormal behavior is defined first, and then all of the other behaviors are defined as normal. The goal of the system is to detect whether the user’s behavior conforms to the defined abnormal behavior [2]. This method can detect the existing intrusion methods, but cannot do anything about new ones. Intrusion detection systems can be divided into network-based intrusion detection systems (NIDS) [3] and host-based intrusion detection systems (HIDS) [4,5], according to different information sources. NIDS focuses on discovering attack information in the network, while HIDS can find abnormal situations in the system logs. Different intrusion detection systems have their advantages and disadvantages. Therefore, it is necessary to deploy different types of intrusion detection systems in different locations in order to monitor as many aspects of the network system as possible [6]. 

According to the principle of intrusion detection technology, it has the ability to detect known intrusion behavior. However, in reality, the intrusion detection system has a high false positive rate and false negative rate. The first step is to set the threshold of the intrusion detection system in order to reduce the false negative rate and enhance the effectiveness of the intrusion detection system; secondly, distributed intrusion detection systems must be widely used. Low thresholds generate a large amount of alert data, while the alerts that are generated by distributed IDS also contain a large number of redundant alerts and false positives. As a result, administrators waste time on unimportant alerts, and it is difficult to find the actual security events that are covered by redundant alerts [7]. Especially under the development trend of large-scale networks and complicated intrusion behavior, the application of large-scale distributed systems in the field of intrusion detection is becoming increasingly widespread. However, the heterogeneity and autonomy of the distributed systems and defects of traditional intrusion detection technology make duplicate or imperfect alert events rampant [8]. For example, for a distributed denial of service (DDoS) attack probably launched by Botnet, which is controlled by its master and formed by a large number of bots, when the host or network system is attacked by thousands of bots, it is difficult to imagine how to handle large amounts of alerts that are generated by IDSs. According to statistics [9,10,11], IDS often produces a large number of alerts in a short time, more than 85% of which are false or irrelevant alerts. Obviously, reducing the redundant alerts that are generated by the intrusion detection system has a certain practical significance in improving the performance of the entire intrusion detection system.

This paper mainly focuses on alert aggregation reduction that is based on attribute similarity and proposes an improved method. While considering that different attacks have different characteristics, the important attributes of different attacks should be different, and the corresponding attribute weights should also be different. In previous studies [12,13,14], the selection of attributes and the setting of attribute weights mostly depended on expert experience. It ignored the objective characteristics of attributes, and the results were easily affected by decision-makers’ lack of sufficient knowledge. Therefore, we propose a method for calculating the attribute weights of specific attack scenarios via combining conditional rough entropy and knowledge granularity. Using the attribute similarity method, the events with certain similarities are aggregated to eliminate the redundant and duplicate alerts and improve the efficiency of alert analysis for network administrators.

The structure of this paper is as follows: Section 2 introduces related work. Section 3 provides the theoretical background and introduces the rough set theory, information systems, and knowledge granularity. Section 4 proposes an alert aggregation method while using an improved attribute weighting algorithm. In Section 5, the experiment is carried out and the experimental results are given. The paper is concluded in Section 6.

## 2. Literature Review

In recent years, network security event aggregation and correlation analysis that are based on the research on the correlation algorithm have gradually become hot spots in the field of network security, and some meaningful results have been produced. Researchers have done a lot of work on the correlation of events and have proposed methods, such as alert correlation based on a sequence [15,16,17], alert correlation based on known scenarios [18], and alert correlation based on attribute similarity [19,20].

• Alert correlation based on a sequence

This type of method determines a correlation that is based on the cause and effect of an alert event. Pre/Post-conditions and graphs are the two most widely used technologies. Hu et al. [15,16] fused the alert information in two dimensions of time and space based on the causality between the various steps of a multi-step attack. This paper proposed a security situation quantification method that was based on attack prediction to assist security administrators in grasping the security status trend of the entire network. In [21], an improved correlation analysis framework, IACF (Intrusion Action Based Correlation Framework), for detecting multi-step attack scenarios is proposed. When compared to our method, the framework uses a new original alert grouping method that is based on intrinsic strong correlation concepts, instead of traditional time windows and hyper-alerts. The pruning algorithm is used to remove redundant actions and action link modes in the session to reduce the impact of false positives in order to find the high stable correlation between actions. However, while the SPA (Sequence Pruning Algorithm) algorithm can greatly reduce false positives, because there is no binary correlation in the SPA algorithm, it also filters out some single-step attacks in intrusion scenarios. In [22], a multi-step attack detection model that is based on SGMS (Smart Grid Monitoring System) alerts is proposed. In this model, an alert graph is constructed through IP correlation, and the child nodes of the alert graph are aggregated. Denoising is then performed through negative causally-related pruning and non-cascading events. Finally, an attack chain and visual attack graph are formed. While the model has a good performance, it requires a small amount of prior knowledge to automatically extract multi-step attack events and demonstrate the trajectories among IPs. Our method does not require any prior knowledge. It only discovers knowledge that is based on the characteristics of the data itself.

The sequence-based correlation analysis method can fully explore the impact of the event on the correlation relationship, but not all relationships can accurately reflect the attack intention. The results of this method may contain many false alerts. This is especially common when the logical predicates are poorly configured, or the quality of sensor alerts is low. In addition, the cost of building a knowledge base while using this method is high, and the tolerance for false negatives is low.

• Alert correlation based on known scenarios

This method correlates different alerts by comparing specific system behaviors with predefined scenarios in a knowledge-based system. In [23], an alert model that was related to the background of the problem was established. The main objective of the established correlation model was to detect attack scenarios and generate early alerts, showing the actions of the next intruder in the target network. Its contribution lies in proposing a new framework for extracting alerts from IDS. The framework changes a series of alerts into a set of alerts and then serializes them in the form of super alerts in the prediction of the next attack. In [18], early warnings are correlated based on a knowledge base and the related likelihood. An attack path construction algorithm is proposed for obtaining the attack path of the specified target IP, and an alert correlation graph is constructed to correlate the alerts to a specific range and then merge them based on the alert type. In [24], an effective false positive recognition model that was based on the gradient boosting tree model was proposed. This article designs a graph-based method to analyze and extract important features from aggregation and correlation to identify the false alerts. Different from our feature selection method, this paper proposes a bidirectional recursive feature elimination method combined with the random forest algorithm. However, the imbalance problem of categories cannot be effectively solved because the model only uses a simple integration method.

The correlation method that is based on known scenarios can effectively correlate predefined attack scenarios, but it relies heavily on its knowledge base. It is difficult to enumerate all possible attack scenarios in advance and create a useful knowledge base within a reasonable time frame.

• Alert correlation based on attribute similarity

The similarity-based alert correlation method associates different alerts by defining and using alert similarity. The main assumption of this approach is that similar alerts have the same root cause or a similar impact on the system being monitored, and similarity is evaluated by comparing the predefined features. This method has advantages in early alert correlation research. It can better aggregate known attack types, and the algorithm has high calculation efficiency and good real-time performance. At the same time, it is not too complicated, so it can be implemented in a variety of different systems. In addition, it has proven effective in reducing the total number of alerts for alert correlation and aggregation processes. When compared with the above two methods, this method cannot describe the attack path. It primarily aggregates alerts for the same event.

The theory of the attribute similarity method is based on clustering, which aggregates and classifies events that meet a certain similarity. Researchers have conducted many studies on alert reduction methods based on attribute similarity to eliminate redundancy or duplication and improve the efficiency of network administrator alert analysis. Valdes and Skinner [25,26] first proposed an alert aggregation method that was based on attribute similarity. This method predefines the similarity function between various attributes of the alert data, such as the IP address, port, and time attribute, in order to calculate the similarity value. Each attribute is assigned a different weight to calculate the overall similarity value. Finally, the total similarity value is compared with a pre-set threshold to determine whether the alerts should be aggregated. The advantage of this method is that it can effectively aggregate similar alerts, and the calculation is relatively simple. Cuppens et al. [13] used an expert system, in which expert rules specified similarity measures. These rules specify similarities between specific attributes in a universe: classification similarity (the type of attack), temporal similarity, and source-target similarity. After measuring similarity, alert instances are assigned to global alerts (or clusters), thereby avoiding alert redundancy. However, this method relies too much on expert experience and might be subjective. Julisch [27] gives a description of the similarity between alerts based on a defined classification method. In some taxonomy, the closer the attributes are, the more similar the two alerts are. This paper proposes an attribute-oriented inductive data mining heuristic algorithm. In addition, Julisch proved that the future alert load can be reduced by more than 90%. Moreover, he points out that the intrusion detection alerts are very homogeneous and repetitive. 

In [28], the structure of intrusion detection alerts is analyzed and an SR-ISODATA algorithm for intrusion detection alert aggregation is proposed. The algorithm is proposed to replace the original algorithm’s Euclidean distance calculation with the average similarity radius and modify the merge segmentation criterion. The attribute weight calculation of the alert data is based on the optimal sequence comparison method, and different attributes are selected for different similarity calculation methods. In [19], a new alert correlation model is proposed by analyzing the characteristics of an alert flow, and the similarity method is used to define the correlation between alerts, without using the pre-defined knowledge and prerequisites. Two steps are introduced in the article to analyze the characteristics of the alert flow, namely, low-level alert analysis and high-level alert analysis. In low-level alert analysis, characteristic measures measure the correlation of similar alert types, and high-level alert analysis is used to discover the correlation between two different types of alerts. Wu et al. [20] applies similarity-based alert correlation technology in the field of CMS (Cyber-Manufacturing System) network physical attack detection. It includes three steps: network alert correlation, physical alert correlation, and network physical alert correlation. In each step, the attributes are defined according to the characteristics of the physical network attack and the CMS environment. Different manufacturing attributes are created and used for similarity-based alert correlation.

There are some limitations to using the alert aggregation reduction method that is based on attribute similarity. First, it is necessary to select an appropriate attribute and similarity calculation function in order to calculate the overall similarity of all attributes. Generally, the selected attributes include source and destination IP addresses, source and destination ports, attack types, timestamps, etc. However, different attack types have different characteristics, and the same attributes cannot express the specificity of an attack. As we all know, different attributes and different similarity calculation functions will have different results. Therefore, it is necessary to select different attribute sets for different attack types and define different attribute similarity functions for different attributes. In addition, the traditional attribute similarity aggregation method does not consider the weight of attributes; it only assigns the same weight to each attribute or relies on expert experience, thus ignoring the objective weight of attributes. However, the weights defined by experts’ experiences are too subjective and arbitrary. If we rely on different experts, we will obtain different weights, and it is difficult to fully reflect the true weights of attributes.

Therefore, the determination of attribute weights is a key issue when using the attribute similarity method for alert aggregation. Wei et al. [29] used gray correlation analysis to determine the importance of alert attributes in classification and then used it as the weight of attributes. However, they assign the same attribute weight to all attack types, while ignoring that the attribute weight might be different, depending on the attack scenario. Yao et al. [30] used the calculation of the dependence of decision attributes on conditional attributes as the basis for judging the importance of attributes in rough set theory. This calculation is applied to historical data, and different weights are set for the corresponding attributes of different attack categories. However, this method cannot exploit the characteristics of the attack well and it only uses a fixed time window. Zang et al. [31] used the same attribute weight determination method as that used in [30]. In addition, they considered that different attacks may have different attack speeds, so they updated the time interval, and the aggregation efficiency was improved.

Inspired by the literature that is presented above, we propose an alert correlation and aggregation method based on attribute similarity. We mainly focus on the improvement of the attribute weight determination method in the aggregation process. We propose a method of attribute selection and attribute weight determination based on conditional rough entropy and knowledge granularity, which can find the difference of attributes in different attack scenarios better and improve the efficiency of alert aggregation.

## 3. Theoretical Background

Professor Pawlak of Warsaw University of Technology in Poland proposed rough set theory in 1981. It is a method of studying incomplete, uncertain knowledge and data for expression, learning, and induction [32,33,34]. Rough sets have been widely used in many fields, such as pattern recognition, data mining, and machine learning, because of its characteristics, especially because it does not require prior knowledge [35,36]. With the development of rough set theory, Zadeh [37] proposed the concept of knowledge granularity in 1996, which is a theory that uses granularity in the process of solving problems. Knowledge granularity is an important part of artificial intelligence and information processing [38,39,40]. A knowledge granule is a group of objects gathered together through the indiscernibility, similarity, and proximity of attributes [41,42].

### 3.1. Rough Set Theory and Information Systems

An information system is a basic description of some information expressions [38]. In general, an information system is defined as I=(U, AT, V, f), and it is also called an approximate space or knowledge base. Where $U=\left\{x_{1}{,x}_{2},\ldots {,x}_{n}\right\}$ is a collection of objects that are finite and not empty (the universe of discourse), *AT* is a set of finite non-empty attributes and *V* is a set of attribute values. For each attribute a∈AT, a set of attribute values $V_{a}$ is associated with the function f: U×AT→V, that is, for each a∈AT, x∈U has $f\;\left(x,\;a\right)\subseteq V_{a}$. A decision table is a special type of information system, which is usually expressed as I=(U, C, D, V, f), where *C* is a finite non-empty set of conditional attributes and *D* is a finite non-empty set of decision attributes, in which C∩D=Φ,C∪D=A.

Rough set theory is based on the classification mechanism. It regards knowledge as the division of finite non-empty object universes. A rough set considers this division as the equivalence relationship between object universes in a specific space. Rough set theory assumes that each object in the universe is related to a certain amount of information (data, knowledge), and this information is expressed by means of some attributes that are used for object description [43]. Objects with the same description are indistinguishable (similar) in the available information. The resulting indistinguishable relationship constitutes the mathematical basis of rough set theory; it divides the universe into several indistinguishable objects, called basic sets, which can be used to build knowledge regarding the real or abstract world.

**Definition** **1.** *For any attribute set A⊆AT, there is an associated indistinguishable relationship R_A_, which is defined as*

(1) $$\mathrm{IND}(A)=\{(x,\;y)\in U\times U|\forall a\in A,f_{a}(x)=f_{a}(y)\}=R_{A}.$$

*If (x, y)∈IND(A), x and y are indistinguishable in the attribute set A, and then the equivalence class of the indistinguishable relation of the attribute set A is defined as ${\left[x\right]}_{A}$ [44]. Obviously, $\mathrm{IND}\left(A\right)=\cap_{a\in A}\mathrm{IND}\left(\left\{a\right\}\right)$. It can be shown that IND(A) is an equivalence relation on U. For any A⊆AT, the relation IND(A) constitutes a partition of U, which is denoted by U/IND(A) or just U/A. That is, $U/A={\left\{[x\right]}_{A}|x\in U\}$ is called information concerning U, where ${\left[x\right]}_{A}=\cup \{x\in U\;|\;\left(x,\;y\right)\in \mathrm{IND}(A)\}=\;\cup \{x\in U\;|\;f(a,\;x)\;=\;f(a,\;y),\;\forall a\in A\}$ is called an equivalence class of x in reference to A.*

Uncertainty is an important problem in information systems. The existing uncertainty measurement methods mainly include knowledge granularity, entropy theory, and roughness. These measures have been successfully applied in many fields. The concept of entropy is derived from classical energy science and it can be used to measure the disorder of a system. The entropy of a system, called information entropy, gives a measure of the uncertainty of its actual structure, as defined by Shannon [45]. It has become a useful mechanism for characterizing various models and applications of uncertainty in many different fields. The concept of rough entropy is introduced in rough sets to measure the rough degree of knowledge more accurately based on information entropy [46]. Rough entropy and information entropy are commonly used measures of uncertainty in information processing [47]. The definition of rough entropy is as follows:

**Definition** **2.** *Let I=(U, AT, V, f) be an information system, for any A∈AT, the rough entropy of A is defined as*

(2) $$E_{r}\left(A\right)=-\overset{m}{\underset{i=1}{\sum }}\frac{\left|X_{i}\right|}{\left|U\right|}\log_{2}\frac{1}{\left|X_{i}\right|}.$$

Rough entropy is used to describe the rough degree of information. The larger the rough entropy, the rougher the information. Obviously, rough entropy has minimum and maximum values: If ${U/R}_{A}={\{\{x}_{1}{\},\{x}_{2}{\},\ldots ,\{x}_{n}\}\}$, the rough entropy of *A* has a minimum value of 0; if ${U/R}_{A}={\{\{x}_{1}{,x}_{2}{,\ldots ,x}_{n}\}\}$, the rough entropy of *A* has a maximum value of $log_{2}\left|U\right|$ [38]. While rough entropy can represent the roughness degree of information, it cannot effectively solve a special information system, such as a decision table. In order to solve this problem, the concept of the conditional rough entropy of information is introduced [48].

**Definition** **3.** *Let I=(U, C, D, V, f) be a decision information system, the equivalent divisions of knowledge P and knowledge Q on U are $U/\mathrm{IND}(P)={\{X}_{1}{,X}_{2}{,\ldots ,X}_{n}\}$, $U/\mathrm{IND}(Q)={\{Y}_{1}{,Y}_{2}{,\ldots ,Y}_{n}\}$. Then, the conditional rough entropy of knowledge Q, relative to knowledge P, is defined as*

(3) $$H\left(Q|P\right)=-\overset{n}{\underset{i=1}{\sum }}\frac{\left|X_{i}\right|}{\left|U\right|}\overset{m}{\underset{j=1}{\sum }}\frac{\left|X_{i}\cap Y_{j}\right|}{\left|X_{i}\right|}\log_{2}\frac{\left|X_{i}\cap Y_{j}\right|}{\left|X_{i}\right|}.$$

This actually explains the use of conditional information entropy to directly define the conditional rough entropy of knowledge. In the above definition, if *P* = *R*, the conditional rough entropy of knowledge *Q*, relative to knowledge *P*, becomes the conditional rough entropy of knowledge *Q*, relative to knowledge *R*, which is the rough entropy of knowledge *Q*.

**Example** **1:** *Let I=(U, C, D, V, f) be a decision information system, in which the field $U={\{u}_{1}{,\;u}_{2}{,\;u}_{3}{,\;u}_{4}{,\;u}_{5}{,\;u}_{6}{,\;u}_{7}{,\;u}_{8}\}$ contains eight objects, the set of conditional attributes $C\;={\{C}_{1}{,C}_{2}{,C}_{3}{,C}_{4}\}$, D is the decision attribute, and the data are normalized and discretized, as shown in Table 1.*

*According to Definition 1, the indiscriminate classification of the universe for condition attributes and decision attributes is*

$$U/D={\{\{u}_{1}{,u}_{3}{,u}_{4}{,u}_{8}{\},\{u}_{2}{,u}_{5}{,u}_{6}{,u}_{7}\}\},$$

$$U/C={\{\{u}_{1}{\},\{u}_{2}{,u}_{6}{\},\{u}_{3}{\},\{u}_{4}{\},\{u}_{5}{\},\{u}_{7}{,u}_{8}\}\}.$$

*From this, the relationship between the equivalent classification sets can be further calculated.*

$$\begin{array}{cc} \left|C_{1}\cap D_{1}\right|=|\{u_{1}\}|\;=1 & |C_{1}\cap D_{2}|=|\Phi |=0\\ |C_{2}\cap D_{1}|=|\Phi |=0 & |C_{2}\cap D_{2}|=|\{u_{2},u_{6}\}|=2\\ |C_{3}\cap D_{1}|=|\{u_{3}\}|=1 & |C_{3}\cap D_{2}|=|\Phi |=0\\ |C_{4}\cap D_{1}|=|\{u_{4}\}|=1 & |C_{4}\cap D_{2}|=|\Phi |=0\\ |C_{5}\cap D_{1}|=|\Phi |=0 & |C_{5}\cap D_{2}|=|\{u_{5}\}|=1\\ |C_{6}\cap D_{1}|=|\{u_{8}\}|=1 & |C_{6}\cap D_{2}|=|\{u_{7}\}|=1 \end{array}$$

*Therefore, while using Equation (3), the conditional rough entropy of decision attribute D, relative to conditional attribute set C, is*

$$H(D|C)=-\overset{n}{\underset{i=1}{\sum }}\frac{\left|C_{i}\right|}{\left|U\right|}\overset{m}{\underset{j=1}{\sum }}\frac{\left|C_{i}\cap D_{j}\right|}{\left|C_{i}\right|}\log_{2}\frac{\left|C_{i}\cap D_{j}\right|}{\left|C_{i}\right|}=0.25.$$

### 3.2. Knowledge Granularity

It is generally believed that knowledge is related to equivalent classification based on the development of rough set theory, which indicates that knowledge is granular. Zadeh [37] proposed Granularity Computing (GrC) in 1996. He identified three basic concepts that emphasized the processes of human cognition, namely, granulation, organization, and causality. “Granulation involves decomposing the whole into parts, organization involves integrating the parts into a whole, and causation involves the association of causes and effects”. Information granularity is mainly used to study the uncertainty of information or knowledge in information systems. Wierman [49] introduced the concept of granularity measurement to measure the uncertainty of information. The concept is the same as the form of Shannon entropy under the proposed axiom definition.

According to the knowledge granularity calculation, ${U/R}_{A}$ is a granular structure and it can be expressed as: $K(R_{A})={\{G}_{R_{A}}{(x}_{1}{),G}_{R_{A}}{(x}_{2}{),\ldots ,G}_{R_{A}}{(x}_{n})\}$. Therefore, the indistinguishable relation *R_A_* is regarded as a granulation method for dividing objects. In particular, the best granular structure on *U* is denoted as $K(\delta )={\{\{x}_{1}{\},\{x}_{2}{\},\ldots ,\{x}_{n}\}\}$, and the worst granular structure is denoted as $K(\omega )={\{\{x}_{1}{,x}_{2}{,\ldots ,x}_{n}\}\}$.

**Definition** **4.** *Definition K = (U, R) is a knowledge base, and R is an equivalent relationship, also known as knowledge. Then, the granularity of knowledge R can be defined as*

(4) $$\mathrm{DG}(R)=\frac{\left|R\right|}{\left|U^{2}\right|}(\mathrm{where}\;|\;R\;|\;\mathrm{represents}\;\mathrm{the}\;\mathrm{base}\;\mathrm{of}\;R\subseteq U\times U).$$

*Let R be the equivalent relationship in the knowledge base K = (U, R), $U/R=\;\{X_{1},\;X_{2},\ldots ,\;X_{n}\}$, then the knowledge granularity can be expressed as: $\mathrm{DG}(R)=\overset{n}{\underset{i}{\sum }}\frac{|X_{i}{|}^{2}}{{\left|U\right|}^{2}}$. Knowledge granularity represents the indistinguishability of the equivalence relation R to knowledge. When $(u,\;v)\;\in {\;X}_{i}$, this means that objects u and v belong to the same equivalence class of the equivalence relation R, and they are therefore indistinguishable under R. From this, it can be known that with the increase of DG (R), the number of objects belonging to the same equivalence class of the equivalence relation R increases, the resolution capability of R decreases, and the knowledge becomes more indistinguishable. Therefore, DG (R) represents the possibility of the R-indistinguishable selection of two objects randomly in U. The greater this possibility, that is, the greater the DG (R), the weaker the resolution of R; otherwise, the resolution is stronger.*

**Definition** **5.** *The discernibility of the equivalence relation R is defined as*

(5) $$\mathrm{Dis}(R)=1-\mathrm{DG}(R)=1-\overset{n}{\underset{i}{\sum }}\frac{{\left|X_{i}\right|}^{2}}{{\left|U\right|}^{2}}.$$

The discernibility of the equivalence relationship *R* represents the ability of *R* to distinguish knowledge. It can also be understood as the importance of the equivalence relationship *R* to classification. The larger the value of Dis(R), the smaller the knowledge granularity of the corresponding equivalence relation *R*, that is, the finer the knowledge division, which means that *R* is more important for classification.

**Example** **2:** (Continued from Example 1) *We continue to use the data in Table 1. In Example 1, we classify the conditional attribute set equivalently, so, according to definition 5, we can calculate the discernibility of the conditional attribute C as*

$$\mathrm{Dis}(C)=1-\mathrm{DG}(C)=1-\sum_{i}^{n}\frac{{\left|C_{i}\right|}^{2}}{{\left|U\right|}^{2}}=1-\frac{1^{2}+2^{2}+1^{2}+1^{2}+1^{2}+2^{2}}{8^{2}}=0.8125.$$

## 4. Proposed Method

### 4.1. Overview of the Aggregation Scheme

This paper proposes an alert aggregation method that is based on security event attribute discovery. The aggregation process includes two decisive steps: the determination of the weight of the important attributes of security events and the aggregation of alerts based on their similarity. The premise of this method is alert data collection and preprocessing.

**Figure 1:**
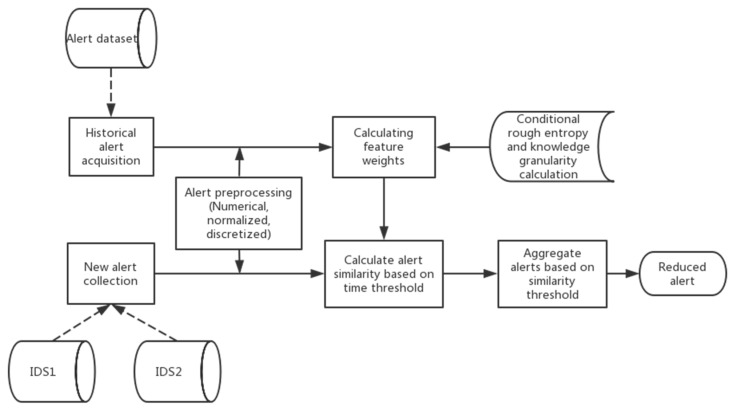
Overview of our alert aggregation model.

The framework obtains alert data and performs pre-processing operations, such as digitization, normalization, and discretization, as shown in Figure 1. We propose an attribute weight calculation method that us based on attack classification to conduct a targeted analysis of attack events and apply this method to historical data to obtain important attribute weights for different attack classifications while combining conditional rough entropy and knowledge granularity. In a real environment, new alerts that are generated by distributed IDS are collected and preprocessed to a uniform format. Subsequently, the attribute similarity value of the alert and the alert within the time window are calculated according to a preset time window threshold. The attributes here are important attributes corresponding to the attack type, and they are obtained by the attribute weight determination method. Finally, the attribute similarity is weighted to obtain the overall similarity of the two alerts. If the value is greater than the preset similarity threshold, the corresponding two alerts can be aggregated. The scheme mainly consists of three parts: alert preprocessing, attribute weight allocation, and alert similarity calculation.

Alert preprocessing: This part includes three processing steps: attribute digitization, normalization, and discretization. Since the alert data contain some non-numeric attributes, such as the protocol type, service type, alert type, etc., in order to facilitate subsequent calculations, the first thing we need to do is digitize the attributes and convert the character attributes into numeric attributes. The function of data normalization is to summarize the statistical distribution of a unified sample and map all of the attributes of the data to the same scale, so as to prevent certain attributes of the data forming a dominant role due to their different dimensions. Rough set theory, as an inductive learning method, can only deal with discrete data. Therefore, we use cluster discretization to convert continuous attributes into discrete attributes before calculating the attribute weights.

Attribute weight distribution: Alerts under different attack types may present different patterns in alert characteristics, but previous studies have ignored this. We propose the use of a combination of conditional rough entropy and knowledge granularity with real historical alert data to extract the important corresponding attributes and attribute weights of different attack classifications in order to accurately reduce redundant alerts.

Calculation of alert similarity: The basic idea of the alert aggregation method that is based on similarity is to calculate the similarity values of important attributes for different types of attacks and then calculate the overall similarity by weighting each attribute. Sufficiently similar alerts will be aggregated into super alerts to reduce the number of duplicate and similar alerts.

### 4.2. Attribute Discretization

Each alert contains discrete attributes, such as the connection duration, number of bytes sent and received, timestamp, and so on. Rough set theory, as an inductive learning method, can only deal with discrete data. Therefore, it is necessary to convert an input dataset with continuous attributes to a dataset with discrete attributes before calculating the attribute weights.

The discretization operation involves grouping the range of continuous data values and dividing them into discrete intervals. Subsequently, different symbols or integer values are used to represent the data values that fall into each interval. Thus, discretization involves two processes: determining the number of classifications *n* and mapping continuous attribute values to n classification intervals. The equal width discrete, equal frequency discrete, and cluster discrete are included as common methods for the discretization of continuity attributes [50]. In this section, we use K-Means cluster discretization to transform the continuous data.

**Figure 2:**
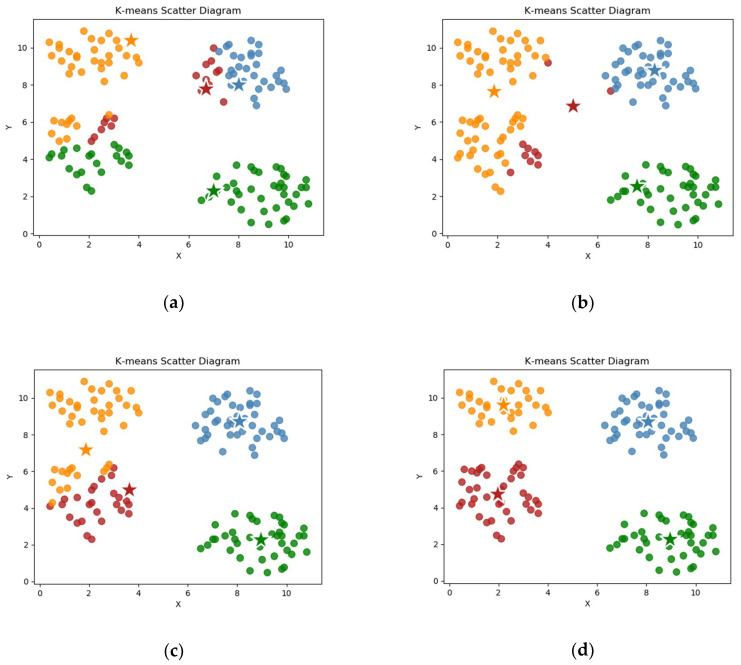
Scatter diagram of the main process of the K-Means clustering algorithm. (**a**) *k* center points are randomly selected, and all sample points are assigned to the nearest cluster; (**b**) the new center point is determined according to the distance, and the sample points are reclassified; (**c**) the center point and classification of the sample points continue to be selected; and, (**d**) the center point no longer changes, and the cluster ends.

The K-Means clustering method remains one of the most popular clustering methods, and it is also suitable to be used to discretize continuously valued variables, because it calculates the continuous distance-based similarity measure to cluster the data points. K-Means is a non-hierarchical partitioning clustering algorithm that operates on a set of data points and assumes that the number of clusters (*k*) to be determined is given. The main idea of the K-Means algorithm is roughly the following: initially, the *k* data points are randomly assigned as the so-called cluster centers. Each data point of a given set is then associated with the closest center, which results in an initial distribution of the cluster. After this initial step, the average of all values in each cluster is calculated as the new cluster center. Each data point is redistributed to the closest center to form a cluster again. The two steps of cluster center selection and sample point division are repeated, until the center point no longer changes, as shown in Figure 2.

In fact, as the continuous attribute discretization operation involves only one variable, it is equivalent to a “one-dimensional” K-Means clustering analysis. One-dimensional cluster discretization includes three processes:

- The elbow method is used to obtain the optimal number *k* of each attribute.
- The clustering algorithm (K-Means algorithm) is used to cluster one-dimensional continuous attributes. The *k* clusters are obtained, so that the intra-cluster distance is the smallest, and the inter-cluster distance is the largest.
- The classification value corresponding to each cluster (similar to this cluster label) is obtained, and the continuous data in the corresponding cluster is then replaced with its cluster label to become the new discrete data.

The cluster discretization method can better discretize continuous data, which is convenient for us in using rough sets and knowledge granularity methods to mine data characteristics and extract data attribute weights.

### 4.3. Attribute Weight Determination

Zhang et al. [31] used the ratio of the cardinality of the set of positive regions to the cardinality of the set of universes to determine the importance of conditional attributes for decision attributes. However, the cardinality of the set of positive regions is the same, but the set of equivalence relations is different when the two attributes are equivalently classified. For example, in the field U={1, 2, 3, 4, 5, 6, 7, 8, 9}, let X={1, 3, 4, 5, 7, 8}, and let $R_{1}$ and $R_{2}$ be equivalent relations that are defined by *U*, and then assuming that the equivalent divisions that they produce are

$${U/R}_{1}=\left\{\{1,3,4,7\},\{2,5,6\},\{8,9\}\right\}$$

$${U/R}_{2}=\{\{1,7\},\{3\},\{4\},\{2,5,6\},\{8,9\}\}.$$

The positive region of *X*, with respect to $R_{1}$ and $R_{2}$, will be

$$\underline{R_{1}}\left(X\right)={\left\{x|[x\right]}_{R}\subseteq X\}=\left\{1,3,4,7\right\}=\underline{R_{2}}\left(X\right),$$

where $\underline{R}\left(X\right)$ is the lower approximation of *R* on *X*. And the importance of the knowledge characteristics will be

$${\mathrm{Sig}}_{R_{1}}\left(X\right)={\mathrm{Sig}}_{R_{2}}\left(X\right)={|\mathrm{POS}}_{R_{1}}\left(X)|/|U\right|={|\mathrm{POS}}_{R_{2}}\left(X)|/|U\right|=\frac{4}{9}.$$

The attribute importance of $R_{1}$ and $R_{2}$, calculated in this way is the same, but the equivalent division of these two attributes is obviously different. According to the analysis of the above examples, it is shown that the attribute weight that is calculated by this method is inaccurate.

In addition, most of the current alert aggregation methods that are based on similarity of attributes use the same set of attributes to calculate the similarity of different attack types. However, different attacks have different characteristics, and the same set of attributes is not targeted. Therefore, the important attributes of the attack should be different for different attack scenarios. We combined conditional rough entropy and knowledge granularity calculation to solve the above problems. For different classifications of decision attributes, we used the knowledge roughness that was expressed by conditional rough entropy and knowledge granularity to define the relationship between different knowledge attributes and decision attributes. The change in the roughness of knowledge before and after the existence of each attribute indicates its ability to classify different decision attribute values, which is defined as the degree of discrimination of knowledge attributes. When a condition attribute has a greater degree of discrimination for decision attributes, that is, the existence of the attribute can classify the objects well, which means that the attribute is more meaningful for decision attributes. Therefore, we should determine the weight according to the classification ability of the condition attribute.

For a given information system, we need to evaluate its roughness or uncertainty for the target object or target decision. The roughness of the rough set monotonically decreases with the decrease of the granularity of knowledge, which is consistent with people’s cognitive intuition. However, many practical examples show that the roughness of a rough set will not change when the knowledge granules that belong to the positive or negative domain of a set are subdivided. Moreover, when the knowledge particles belonging to a set boundary are subdivided, their roughness may not change, which is inconsistent with people’s cognitive intuition. In rough sets, rough relational databases, and information systems, an uncertainty measure, called rough entropy, has been proposed to calculate the roughness of knowledge to overcome this problem. The combination of roughness and knowledge granularity can more comprehensively describe the rough degree of knowledge. The smaller the roughness of knowledge, the larger the average amount of information it provides, according to the meaning of the roughness of knowledge. Subsequently, its average uncertainty and randomness will be smaller, and its rough entropy should be smaller. Based on rough entropy, conditional rough entropy for decision information systems is proposed. Conditional rough entropy can represent the rough entropy of decision attributes, relative to conditional attributes, namely, roughness. In Definition 2, the conditional rough entropy H(Q|P) represents the average uncertainty that still exists for knowledge *Q*, when knowledge *P* is known. 

While considering a given decision information system I=(U, C, D, V, f), *P* and *Q* are the set of equivalent relations on *U*, and their equivalent divisions on *U* are $U/\mathrm{IND}(P)={\{X}_{1}{,X}_{2}{,\ldots ,X}_{n}\}$, $U/\mathrm{IND}(Q)={\{Y}_{1}{,Y}_{2}{,\ldots ,Y}_{m}\}$. According to Definition 3, we can obtain the conditional rough entropy H(Q|P) of knowledge *Q*, relative to knowledge *P*. However, we hope to obtain the rough degree of different equivalence classifications, with respect to conditional attributes, in the equivalence classification of decision attributes. Therefore, we make a more precise and detailed division of H(Q|P). We obtain the rough degree of knowledge P for each equivalent class $Y_{i}$ of knowledge *Q*, which is the relative condition rough entropy.

**Definition** **6.** *Relative to knowledge P, the relative conditional rough entropy of the equivalent classification Y_i_ of knowledge P is defined as*

(6) $${R(Y}_{i}\left|P\right)=-\overset{n}{\underset{j=1}{\sum }}\frac{{|X}_{j}\cap Y_{i}|}{\left|U\right|}\log_{2}\frac{1}{{|X}_{j}\cap Y_{i}|}.$$

In Definition 5, the discernibility of the equivalence relationship *R* represents the classification ability of *R* for the data. However, it only shows the ability of *R* to distinguish all of the categories of decision attributes and it cannot distinguish the classification ability of the equivalent relationship *R* for different categories. We introduce the concept of relative discernibility in order to calculate the importance of the equivalence relationship, relative to the specific classification of the decision attribute.

**Definition** **7.** *The relative discernibility of knowledge P to the equivalent classification Y_i_ of knowledge Q is defined as*

(7) $${\mathrm{DisR}}_{Y_{i}}\left(P\right)=1-\overset{t}{\underset{z}{\sum }}\frac{|Z_{z}{|}^{2}}{|U{|}^{2}}Z=\left\{X_{j}{|X}_{j}\cap Y_{i}\neq \Phi \right\}=\left\{Z_{1},Z_{2},\ldots Z_{t}\right\}.$$

Knowledge granulation and entropy theory are the two main methods for studying the uncertainty of information systems. Knowledge granulation can be used to characterize the roughness of the knowledge structure. The finer the knowledge structure, the smaller the knowledge granulation. The rough entropy of knowledge decreases with the fine division of classes. According to definition 5, the greater the granularity of knowledge, the less distinguishable the knowledge, and the weaker the classification ability. Because the conditional rough entropy increases with the increase of knowledge granularity, the classification ability of knowledge should be inversely proportional to the conditional rough entropy. From Definition 6, we can obtain the relative condition rough entropy of different equivalence divisions of the decision attributes, relative to the equivalence relation *R*. Combined with the relative discernibility of the equivalence relation, the relative knowledge attribute discernibility of the equivalence relation can be defined.

**Definition** **8.** *Relative to the equivalent classification Y_i_ of knowledge Q, the relative knowledge attribute discernibility of knowledge P is*

(8) $${\mathrm{KFDisR}}_{Y_{i}}\left(P\right)=\frac{{\mathrm{DisR}}_{Y_{i}}\left(P\right)}{10^{{R(Y}_{i}\left|P\right)}}.$$

Let *I* = (*U*, *A*) be an information system, then C⊆A is a subset of conditional attributes, c∈C is an attribute. Because the importance of each condition attribute is different, it is necessary to determine the importance of each attribute. In the rough set, the idea is to remove an attribute first, and then consider how the classification will change without the attribute. If the corresponding classification change is relatively large after this attribute is removed, the intensity of the attribute is large, that is, the importance is high; otherwise, the intensity of the attribute is small, and the importance is low. According to this feature, the ability of attribute *c* to classify a finite non-empty set *U* can be understood as the degree to which the relative knowledge attribute discernibility of set *U* increases (decreases) after (decreasing) attribute *c* is added to attribute set *C*. The more the existence of attribute *c* changes the relative knowledge attribute discernibility of the non-empty set, the stronger the classification ability of the finite non-empty set *U* in the attribute *C*. Therefore, we define the relative attribute importance of attribute *c* as follows.

**Definition** **9.** *Let I=(U, C, D, V, f) be a decision information system, then $Y=U/D\;=\;\left\{{\;Y}_{1}{,\;Y}_{2},\ldots {,\;Y}_{m}\right\}$ is the equivalent classification of the universe U under the decision attribute D. For all condition attributes c∈C, there exist $X=U/C=\left\{X_{1},X_{2},\ldots X_{n}\right\}$ and $Z=U/\left(C-c_{j}\right)=\left\{Z_{1},Z_{2},\ldots ,Z_{t}\right\}$. Afterwards, the relative attribute importance of attribute c∈C is defined as*

(9) $${\mathrm{Sign}}_{Y_{i}}\left(c\right)={\mathrm{KFDisR}}_{Y_{i}}\left(C\right)-{\mathrm{KFDisR}}_{Y_{i}}(C-\left\{c\right\}).$$

Definition 9 can be understood in the following way. When attribute *c* is added to the set C−{c}, the indistinguishability of the equivalence relationship increases, and the number of sets divided into equivalence classes increases, when compared to the original set. Consequently, the discernibility is increased. At the same time, the conditional rough entropy is reduced, the accuracy of the data is improved, and the equivalence division is more accurate. Therefore, we believe that the classification ability of equivalence relations can be improved after the addition of attribute *c*.

Finally, according to the different classifications of decision attributes, the weight *W*(*c*) of condition attributes c∈C is calculated, as

(10) $$W(c)=\frac{{\mathrm{Sign}}_{Y_{i}}\left(c\right)}{\sum_{c\in C}{\mathrm{Sign}}_{Y_{i}}\left(c\right)}.$$

Algorithm 1 shows the steps of the attribute weighting algorithm, which combines conditional rough entropy and knowledge granularity calculation. The condition attribute weights corresponding to different decision attribute values in the decision table are determined according to the influence of the presence or absence of corresponding condition attributes in the roughness of knowledge.
**Algorithm 1:** Method to Determine Condition Attribute Weights**Input:** the knowledge base K=(U, R), R=C∩D;**Output:** the weight of the condition attributes, $W_{i}\left(c\right)=\left\{w\left(c_{1}\right),\ldots ,w\left(c_{j}\right),\ldots ,w\left(c_{n}\right)\right\}$;1:  compute the equivalence class ${\left[X\right]}_{C}={\{X}_{1}{,\ldots ,X}_{j}{,\ldots X}_{n}\}$, ${\left[X\right]}_{D}={\{D}_{1}{,\ldots ,D}_{i}{,\ldots ,D}_{m}\}$2:  **for**
*i* = 1 to *m*
**do** 3:    compute the relative knowledge attribute discernibility ${\mathrm{KFDisR}}_{D_{i}}\left(C\right)$4:    **for**
*j* = 1 to *n*
**do** 5:       compute the equivalence class ${\left[X_{j}\right]}_{\left(C-c_{j}\right)}=U/\left(C-c_{j}\right)$6:       compute the relative knowledge attribute discernibility ${\mathrm{KFDisR}}_{D_{i}}\left(C-c_{j}\right)$7:       compute the relative attribute importance of conditional attribute $c_{j},$
${\mathrm{Sign}}_{D_{i}}\left(c_{j}\right)$8:    **end for**9:    computer the condition attribute weight $W_{i}\left(c\right)$10:  **end for**

**Example** **3:** *We continue to calculate the data shown in Table 1. First, each condition attribute $c_{i}$ is removed from the condition attribute set C, and then the equivalent classification of the universe is calculated by the corresponding equivalence relation:*

$$\begin{array}{c} U/(C-\left\{c_{1}\right\})=\left\{\left\{u_{1}\right\},\left\{u_{2}{,u}_{6}\right\},\left\{u_{3}\right\},\left\{u_{4}\right\},\left\{u_{5}{,u}_{7}{,u}_{8}\right\}\right\},\\ U/(C-\left\{c_{2}\right\})=\left\{\left\{u_{1}{,u}_{3}{,u}_{7}{,u}_{8}\right\},\left\{u_{2}{,u}_{6}\right\},\left\{u_{4}\right\},\left\{u_{5}\right\}\right\},\\ U/(C-\left\{c_{3}\right\})=\left\{{\{u}_{1}{\},\{u}_{2}{,u}_{4}{,u}_{6}{\},\{u}_{3}{\},\{u}_{5}{\},\{u}_{7}{,u}_{8}\}\right\},\\ U/(C-\left\{c_{4}\right\})=\left\{\left\{u_{1}\right\},\left\{u_{2}{,u}_{5}{,u}_{6}\right\},\left\{u_{3}\right\},\left\{u_{4}\right\},\left\{u_{7}{,u}_{8}\right\}\right\}. \end{array}$$

*According to Definition 6, we can calculate the relative conditional rough entropy of the equivalent classifications D_1_ and D_2_ of the decision attribute D, relative to the conditional attribute C, as*

$$\begin{array}{cc} R\left(D_{1}|C\right) & =-\overset{n}{\underset{j=1}{\sum }}\frac{\left|C_{j}\cap D_{1}\right|}{\left|U\right|}\log_{2}\frac{1}{\left|C_{j}\cap D_{1}\right|}\\ & =-\left(\frac{1}{8}\log_{2}\frac{1}{1}+0+\frac{1}{8}\log_{2}\frac{1}{1}+\frac{1}{8}\log_{2}\frac{1}{1}+0+\frac{1}{8}\log_{2}\frac{1}{1}\right)=0,\\ {R(D}_{2}\left|C\right) & =-\overset{n}{\underset{j=1}{\sum }}\frac{\left|C_{j}\cap D_{2}\right|}{\left|U\right|}\log_{2}\frac{1}{\left|C_{j}\cap D_{2}\right|}=-\left(0+\frac{2}{8}\log_{2}\frac{1}{2}+0+0+\frac{1}{8}\log_{2}\frac{1}{1}+\frac{1}{8}\log_{2}\frac{1}{1}\right)\\ & =0.25. \end{array}$$

*The relative conditional rough entropy of the equivalent classifications D_1_ and D_2_ of the decision attribute D, relative to the conditional attribute $C-\;\left\{c_{1}\right\}$, is calculated as*

$$R\left(D_{1}|\left(C-\left\{c_{1}\right\}\right)\right)=-\overset{n}{\underset{j=1}{\sum }}\frac{\left|X_{j}\cap D_{1}\right|}{\left|U\right|}\log_{2}\frac{1}{\left|X_{j}\cap D_{1}\right|}=0,$$

$$R\left(D_{2}|\left(C-\left\{c_{1}\right\}\right)\right)=-\overset{n}{\underset{j=1}{\sum }}\frac{\left|X_{j}\cap D_{2}\right|}{\left|U\right|}\log_{2}\frac{1}{\left|X_{j}\cap D_{2}\right|}=0.5.$$

*While using Equation (7), the relative discernibility of the conditional attribute set C and the equivalent classifications D_1_ and D_2_ of the decision attribute D are calculated as*

$$\begin{array}{c} Z_{1}=\left\{C_{j}{|C}_{j}\cap D_{1}\neq \Phi \right\}=\left\{\left\{u_{1}\right\},\left\{u_{3}\right\},\left\{u_{4}\right\},\left\{u_{7}{,u}_{8}\right\}\right\},\\ {\mathrm{DisR}}_{D_{1}}\left(C\right)=1-\overset{t}{\underset{z}{\sum }}\frac{|Z_{z}{|}^{2}}{|U{|}^{2}}=1-\frac{1^{2}+1^{2}+1^{2}+2^{2}}{8^{2}}=0.8906,\\ Z_{2}=\left\{C_{j}{|C}_{j}\cap D_{2}\neq \Phi \right\}=\left\{\left\{u_{2}{,u}_{6}\right\},\left\{u_{5}\right\},\left\{u_{7}{,u}_{8}\right\}\right\},\\ {\mathrm{DisR}}_{D_{2}}\left(C\right)=1-\overset{t}{\underset{z}{\sum }}\frac{|Z_{z}{|}^{2}}{|U{|}^{2}}=1-\frac{2^{2}+1^{2}+2^{2}}{8^{2}}=0.8594. \end{array}$$

*At the same time, the relative discernibility of the conditional attribute set $C-\;\left\{c_{1}\right\}$ for the equivalent classifications D_1_ and D_2_ of the decision attribute D are*

$$\begin{array}{c} Z_{1}=\left\{C_{j}{|C}_{j}\cap D_{1}\neq \Phi \right\}=\left\{\left\{u_{1}\right\},\left\{u_{3}\right\},\left\{u_{4}\right\},\left\{u_{5}{,u}_{7}{,u}_{8}\right\}\right\},\\ {\mathrm{DisR}}_{D_{1}}(C-\left\{c_{1}\right\})=1-\overset{t}{\underset{z}{\sum }}\frac{|Z_{z}{|}^{2}}{|U{|}^{2}}=1-\frac{1^{2}+1^{2}+1^{2}+3^{2}}{8^{2}}=0.8125,\\ Z_{2}=\left\{C_{j}{|C}_{j}\cap D_{2}\neq \Phi \right\}=\left\{\left\{u_{2}{,u}_{6}\right\},\left\{u_{5}{,u}_{7}{,u}_{8}\right\}\right\},\\ {\mathrm{DisR}}_{D_{2}}\left(C\right)=1-\overset{t}{\underset{z}{\sum }}\frac{|Z_{z}{|}^{2}}{|U{|}^{2}}=1-\frac{2^{2}+3^{2}}{8^{2}}=0.7969. \end{array}$$

*Finally, we can obtain the relative attribute importance of the conditional attribute $c_{1}$ according to the above calculation and Equation (9), as follows:*

$$\begin{array}{c} {\mathrm{Sign}}_{D_{1}}{(c}_{1})={\mathrm{KFDisR}}_{D_{1}}\left(C\right)-{\mathrm{KFDisR}}_{D_{1}}(C-\left\{c_{1}\right\})=\frac{0.8906}{10^{0}}-\frac{0.8125}{10^{0}}=0.0781,\\ {\mathrm{Sign}}_{D_{2}}{(c}_{1})={\mathrm{KFDisR}}_{D_{2}}\left(C\right)-{\mathrm{KFDisR}}_{D_{2}}(C-\left\{c_{1}\right\})=\frac{0.8594}{10^{0.25}}-\frac{0.7969}{10^{0.5}}=0.2313. \end{array}$$

*In the same way, we can obtain the relative attribute importance of the conditional attributes $c_{2}$, $c_{3}$, and $c_{4}$, and calculate the attribute weight values of each conditional attribute for different categories of the decision attribute D, as shown in Table 2.*

### 4.4. Alert Similarity Calculation

We calculated the corresponding important attributes and their weights according to different attack classifications. In order to aggregate similar alerts, we also need to calculate the similarity value of each important attribute between the two alerts and weight the total similarity. The two alerts are aggregated if the total similarity of the two alerts is greater than the set threshold. It should be noted that we only need to forcibly reduce the alerts whose total similarity is greater than the threshold for a certain period of time, so the setting of the time threshold is necessary. Therefore, we use sliding time windows to slice alert sequences and aggregate alerts within the same time window. As we have performed cluster discretization preprocessing on the data, and the data values of the same interval have been classified into one category, we define the similarity function of the attribute $c_{i}$ as

(11) $$S_{i}\{\begin{array}{cc} 0 & the\;two\;alert\;attributes\;c_{i}\;have\;different\;values\\ 1 & the\;two\;alert\;attributes\;c_{i}\;have\;the\;same\;value \end{array}.$$

And we compute total similarity of two alerts as

(12) $$S_{\mathrm{total}}=\overset{n}{\underset{i=1}{\sum }}w_{i}*S_{i},$$

where i is the alert attribute index, *n* is the total number of alert attributes, $w_{i}$ is the weight of the *i*-th important attribute of the alert, $S_{i}$ is the attribute similarity of the attribute $c_{i}$ between the two alerts, and $S_{total}$ is the total similarity value of the two alerts. Once the total similarity between two alerts in the same time interval is greater than a set threshold, the latter is deleted.

## 5. Experiment

### 5.1. Experiment Dataset

There are currently some intrusion detection datasets, such as DARPA 98, KDD 99, and ISC 2012, etc., which have been used by researchers to evaluate the performance of their proposed intrusion detection and intrusion prevention methods. However, in [51], it is shown that many of these datasets are outdated and unreliable. Some of these datasets lack traffic diversity, some datasets do not cover various attacks, while others anonymize packet information, have payloads that do not reflect the current trends, or they lack feature sets and metadata.

The Canadian Institute for Cybersecurity provides an updated dataset, called CIC-IDS 2018 [51], which contains the latest threats and features and represents threats that are not addressed by the old dataset. It covers all eleven necessary standards, with common updated attacks, such as DoS, DDoS, Brute Force, XSS, SQL Injection, Infiltration, Port scan, and Botnet. This dataset uses the CICFlowMeter software, published by the Canadian Institute for Cybersecurity, to extract and calculate more than 70 network traffic characteristics for all benign and attack traffic. CICFlowMeter is a network traffic flow generator, written in Java, which provides greater flexibility in selecting features to calculate, adding new ones, and gaining a better control of the duration of the flow timeout. The output of the application is in a CSV file format, with six columns that are labeled for each flow, namely, Flow ID, Source IP, Destination IP, Source Port, Destination Port, and Protocol, and more than 70 network traffic features.

At the same time, the dataset also has some shortcomings. One of the shortcomings is the huge amount of data, with five days of traffic information from The Canadian Institute for Cybersecurity in eight files, each of which has hundreds of thousands of records. As a result, the dataset contains many redundant records, which makes it harder to detect attacks. Therefore, we apply the proposed alert aggregation algorithm to this dataset to evaluate the efficiency of the algorithm.

In addition, we also use the DARPA 98 dataset, which has been widely used in order to better evaluate the efficiency of the algorithm. The DARPA 98 dataset is collected and constructed by the Lincoln Laboratory Information System Technology Group, and it is widely used for the training and testing of the alert correlation system. This dataset collects data from seven weeks, including over millions of attacks events. There are four types of attack events that were collected by the dataset: DDOS, Probes, R2L, and U2R. We selected some data on each attack type for experiments to verify the effectiveness of the aggregation method.

### 5.2. Experimental Setup

We use the CICIDS 2018 dataset and the DARPA dataset for the experiments. The CICIDS 2018 dataset covers seven types of attacks: DoS, DDoS, Brute Force, XSS, SQL Injection, Infiltration, and Botnet. Moreover, the DARPA 98 dataset contains four categories of attacks: DDOS, Probes, R2L, and U2R. In the face of extremely large amounts of data, we used data sampling to process them. Such errors will not be very high, which greatly improves the processing efficiency and processing success rate. We randomly extract a part of the records for each attack type in the two datasets and then divide the data for each type of attack into two parts. One part of them is regarded as real historical data, which uses our proposed attribute weight determination algorithm, which combines conditional rough entropy and knowledge granularity to extract attribute weights. Moreover, our scheme was used to aggregate alerts in the other part.

The experimental operating environment is Intel Core i5-7500 CPU 3.40GHz, and the memory is 8 GB. The software environment is as follows: the operating system is Microsoft Windows 10, the experimental program is written in Python, and the development environment is python 3.7.3. PyCharm is the development tool. As an interpreted, object-oriented, and dynamic data type high-level programming language, Python is widely used in data science. Python provides a rich feature set to perform these tasks in collecting data, cleaning datasets, extracting important features, building machine learning models, and visualizing results while using graphics.

### 5.3. Experimental Results

In this section, we first give the feature weight distribution results of the CIC-IDS 2018 dataset based on the definition in Section 4.3. For each different attack category, we set its important attribute composition, according to the characteristics of the data itself, and calculate the corresponding attribute weight. Here, we choose five representative attack types, and Table 3 shows the results of attribute weight distribution.

The alert aggregation rate is defined as the evaluation standard in the experimental analysis in order to measure the effect of alert aggregation. Assuming that the number of original alerts is *N*, and the number of remaining alerts after aggregation is *n*, the alert aggregation rate can be expressed as

(13) $$\delta =\frac{N-n}{N}.$$

**Figure 3:**
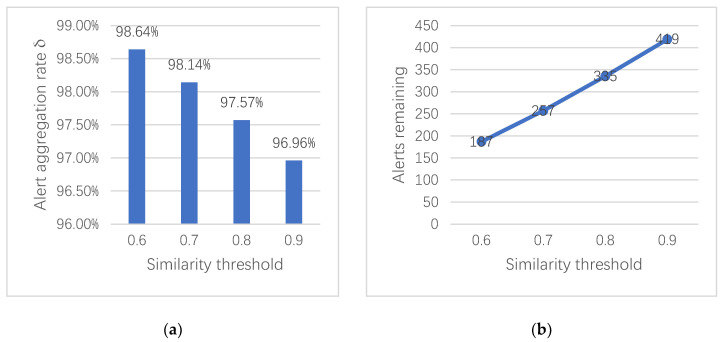
Reduced results of 13796 DoS GoldenEye attack records, with a time threshold of 2 s. (**a**) The change of aggregation rate under different similarity values. (**b**) The number of remaining alert record changes with different similarity values.

The alert aggregation rate *δ* is used to indicate the efficiency of the aggregation algorithm to reduce the redundant and duplicate data. Because the dataset is too large, in order to simplify the experiment, we randomly select records of various types of attack traffic in the dataset over a period of time. Here, we take the DoS GoldenEye attack as an example and select 13796 attack records for alert aggregation. The attack attribute weight distribution in Table 3 is used to calculate the similarity value between alerts within a specified time window, according to Section 4.4. The setting of the time window is based on the actual attack situation, where it is set to 2 s. We use four different similarity thresholds of aggregate alerts to compare the aggregation effects, and Figure 3 shows the results of duplicated alert reduction.

**Figure 4:**
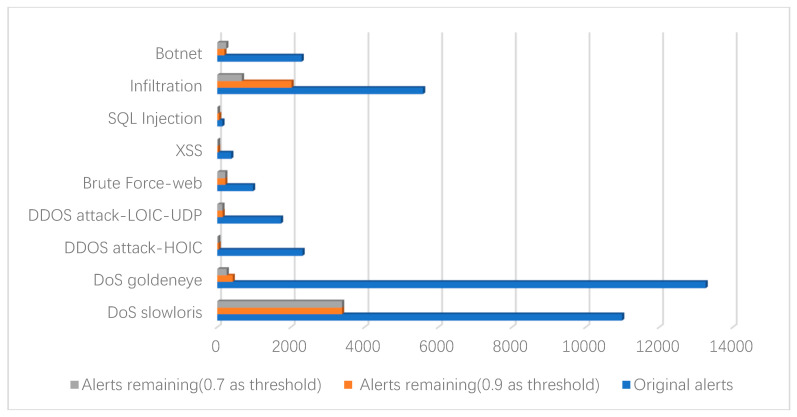
Result of alert aggregation.

In our scheme, the aggregation rate decreases with the increase of the similarity threshold, as can be seen from Figure 3. When the similarity threshold is 0.6, the experiment reaches the optimal aggregation rate of 98.64%. The average aggregation rate of the four similarity thresholds is approximately 97.83%. However, when the similarity threshold is set too low, non-redundant data may be removed. A higher similarity threshold should be set according to the specific situation in order to ensure the integrity of the alert and reduce the loss of information during the aggregation process. A suitable similarity threshold can effectively eliminate duplicate alerts and provide higher quality data for the next data fusion layer. Finally, we applied the aggregation method to a part of the records of the seven attack types and obtained the aggregation results that are shown in Figure 4.

From the data chart illustrated in Figure 4, we can see that the intrusion detection system collects a large number of duplicate and redundant alerts. Moreover, real attack events are hidden in it, which causes huge difficulties in network security management. In the experiment, we applied the proposed attribute weight determination algorithm to the dataset, which can, by itself, discover the characteristics of the data well. At the same time, the alerts are aggregated through attribute similarity calculations. Subsequently, by setting a higher attribute similarity threshold, it can effectively remove redundant duplicate alerts, while ensuring acceptable information loss, thus providing accurate analysis data for the next data fusion process. 

We choose three similarity-based alert correlation methods for comparison experiments since our method is based on attribute similarity. These three methods have become representative methods in recent years. They are the no-weight method, scheme 1 [29] and scheme 2 [31], which are different from our method in terms of the attribute weight determination and aggregation method. The no-weight method assigns the same weight to each attribute, and its aggregation method is the same as ours. Scheme 1 [29] and scheme 2 [31] adopt different attribute weighting methods and aggregation methods. Therefore, these three methods and our method have comparative significance. We apply these three methods and our method to the CIC-IDS 2018 dataset and the DARPA 98 dataset and compare the efficiency of alert aggregation, as shown in Table 4.

**Figure 5:**
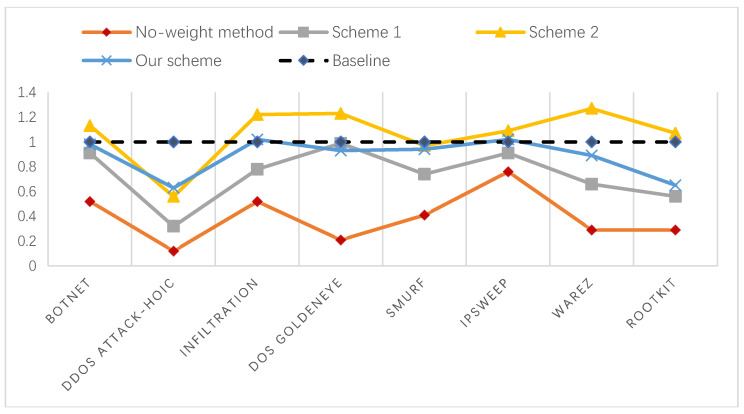
Comparison of the soundness of different aggregation algorithms.

All of the schemes in Table 4 have a similar consideration of the alert’s feature quantity and they exclude the irrelevant attribute. Our scheme and the scheme in [31] both employ a classification-based approach to reduce duplicate alerts, while the scheme in [29] does not consider this aspect. The ratios of the four schemes are approximate for the aggregation rate. Overall, our scheme is ahead of the no-weight method approach and scheme 1 [29], but slightly lower than scheme 2 [31], in terms of its reduction efficiency. However, we should also consider the integrity of the information in addition to the aggregation rate. Excessive aggregation can lead to a loss of alerts, which is very dangerous for intrusion detection. We define the soundness *α* of the system to measure the integrity of the alert, as follows:

(14) $$\alpha =\frac{\mathrm{td}}{N-N_{f}},$$

where *td* is the number of alerts that the system actually detected correctly, *N* is the number of original alerts, and $N_{f}\;$is the number of alerts filtered out after aggregation. Subsequently, $N-N_{f}$ represents the number of remaining alerts after aggregation. Soundness *α* is used to measure the correctness of the recommended alerts. We counted the number of true alerts in the dataset and compared the soundness of the different methods, as shown in Figure 5.

According to the definition of soundness, when the value of *α* is greater than 1, the number of remaining alerts after aggregation is less than the number of true alerts, which indicates that there is a loss of information during the aggregation process; when the value of *α* is less than 1, the aggregation is insufficient, so the aggregation rate is low. Therefore, the closer the value of *α* is to 1, the better the alert aggregation effect. Our method is the closest of the four methods to the baseline in terms of the overall trend, as can be seen from Figure 5. Table 4 shows that the aggregation rate of scheme 2 is higher than our method. However, from the perspective of soundness, scheme 2 has certain information loss. It removed some of the alerts that should not be reduced during the aggregation process, which results in inaccurate results. In summary, on the whole, our method can guarantee the aggregation rate and integrity of alerts to a certain extent.

Finally, we compared the time complexity of the four methods, as shown in Table 5, where *m* is the number of condition attributes, *n* is the number of equivalent classifications of decision attributes, and *u* is the total number of alert objects. The similarity-based correlation and aggregation method mainly has two key steps, namely, the determination of attribute weights and alert aggregation. We separately calculated the time complexity of these two steps. In Table 5, the time complexity of the two steps of the four methods is sorted. For the attribute weighting methods, we have: $T_{1}\left(\mathrm{no}-\mathrm{weight}\;\mathrm{method}\right)<T_{1}\left(scheme\;1\right)=T_{1}\left(scheme\;2\right)<T_{1}\left(\mathrm{our}\;\mathrm{scheme}\right)$, where $T_{1}\left(M\right)$ denotes the time complexity of the attribute weighting algorithm *M*. As for the alert aggregation algorithm, there are: $T_{2}\left(\mathrm{no}-\mathrm{weight}\;\mathrm{method}\right)=T_{2}\left(\mathrm{our}\;\mathrm{scheme}\right)<T_{2}\left(scheme\;1\right)<T_{2}\left(\mathrm{scheme}\;2\right)$, where $T_{2}\left(N\right)$ denotes the time complexity of the alert aggregation algorithm *N*. 

While the time complexity of our attribute weighting algorithm is high, we can better find the characteristics of different attack types and flexibly determine their important attributes and weights. On the other hand, the difference between these four aggregation methods is in the determination of the time interval. Scheme 1 uses the time interval dynamic update method and scheme 2 uses the time stamp as a condition attribute for aggregation. Therefore, the time complexity of these two methods is high. Our aggregation algorithm is the same as the no-weight method, with lower time complexity. At the same time, our method has higher aggregation efficiency while considering the aggregation rate and soundness.

## 6. Discussion and Conclusions

This paper proposes an alert data aggregation method to remove redundant duplicate data that were generated by the intrusion detection systems. First, an improved attribute weighting method is proposed, which is combined with conditional rough entropy and knowledge granularity calculation. This method can better find the important attributes of corresponding attacks and measure their attribute weights. Subsequently, the similarity of each two alerts within a set time window is weighted according to the weight calculation result. Finally, alert reduction is performed according to the different set thresholds of similarity. We use the K-Means clustering method to discretize continuous data, and the similarity is determined based on whether the data belong to the same cluster. The experimental results for the CIC-IDS 2018 dataset and the DARPA 98 dataset show the applicability and effectiveness of our proposed method. We compare the experimental results of different schemes, such as the no-weight method, scheme 1 [29], scheme 2 [31], and our proposed method. The comparisons indicate that our method has a better effect. Different from other methods, our method of determining attribute weights is based on attack classification. Our method can select the corresponding important attributes according to the characteristics of the attack itself while considering the different performance of alert attributes in different attack scenarios. Our method has certain flexibility and pertinence, and it is able to more comprehensively discover knowledge and reduce information loss in the process of aggregation to some extent. Of course, our method also has some flaws. The addition of attack classification leads to a higher time complexity of the attribute weighting algorithm. However, our goal is to aggregate alerts, so this weakness can be tolerated when the aggregate results are more accurate. In summary, our scheme can effectively reduce redundant alerts and help network security administrators to find real attacks.

In future work, we hope to apply our scheme to real-world attacks to better test the performance of our scheme. At the same time, we will focus on the next stage of alert correlation, with a view to reconstructing the entire attack scenario.

## Figures and Tables

**Table 1:** A decision information system.

*U*	$C_{1}$	$C_{2}$	$C_{3}$	$C_{4}$	*D*
$u_{1}$	1	2	2	3	a
$u_{2}$	2	3	2	1	b
$u_{3}$	1	1	2	3	a
$u_{4}$	2	3	1	1	a
$u_{5}$	2	3	2	3	b
$u_{6}$	2	3	2	1	b
$u_{7}$	1	3	2	3	b
$u_{8}$	1	3	2	3	a

**Table 2:** Attribute weight assignment.

	*D*	*C*_1_	*C*_2_	*C*_3_	*C*_4_
Relative Attribute Importance	a	0.0781	0.7037	0.125	0
	b	0.2313	0.1054	0.0439	0.2805
Attributes Weights	a	0.0861	0.7760	0.1378	0
	b	0.3498	0.1595	0.0665	0.4242

**Table 3:** Important attributes of different attack categories and their weight distribution.

Label	Attribute Name	Description	Weight
DoS GoldenEye	Flow IAT Min	Minimum time between two flows	0.06473
	Fwd IAT Std	Standard deviation time between two flows	0.07628
	Pkt Len Max	Maximum length of a flow	0.04184
	Init Fwd Win Byts	Number of bytes sent in initial window in the forward direction	0.56814
	Init Bwd Win Byts	Number of bytes sent in initial window in the backward direction	0.23577
	Idle Max	Maximum time a flow was active before becoming idle	0.01323
DDOS attack-LOIC-UDP	Flow Pkts/s	Flow packet rate, that is, number of packets transferred per second	0.029746
	Flow IAT Std	Standard deviation time between two flows	0.029860
	Fwd IAT Std	Standard deviation time between two packets sent in the forward direction	0.720685
	Fwd Pkts/s	Number of forward packets per second	0.219709
Brute Force-web	Bwd Pkt Len Std	Standard deviation size of packet in backward direction	0.029314
	Flow IAT Std	Standard deviation time between two flows	0.047338
	Flow IAT Max	Maximum time between two flows	0.632954
	Init Fwd Win Byts	Number of bytes sent in initial window in the forward direction	0.290394
SQL Injection	Fwd Pkt Len Max	Maximum size of packet in forward direction	0.110489
	Fwd Pkt Len Std	Standard deviation size of packet in forward direction	0.114238
	Bwd Pkt Len Std	Standard deviation size of packet in backward direction	0.010134
	Bwd Pkts/s	Number of backward packets per second	0.168801
	Init Fwd Win Byts	Number of bytes sent in initial window in the forward direction	0.517399
	Init Bwd Win Byts	Number of bytes sent in initial window in the backward direction	0.078939
Infiltration	Dst Port	Destination port number	0.203669
	Fwd Pkt Len Std	Standard deviation size of packet in forward direction	0.135869
	Bwd Pkt Len Min	Minimum size of packet in backward direction	0.253720
	Pkt Len Var	Minimum inter-arrival time of packet	0.092476
	Init Fwd Win Byts	Number of bytes sent in initial window in the forward direction	0.125480
	Fwd Act Data Pkts	Number of packets with at least 1 byte of TCP data payload in the forward direction	0.188786

**Table 4:** Comparison of aggregation rates with other alert aggregation schemes in relation to different datasets.

Datasets	Attack Category	Number of Original Alerts	Alert Aggregation Rate			
			No-Weight Method	Scheme 1 [29]	Scheme 2 [31]	Our Scheme
CIC-IDS 2018	Botnet	2292	79.89%	88.43%	90.71%	89.27%
	DDOS attack-HOIC	2315	88.55%	95.81%	97.62%	98.79%
	Infiltration	5587	76.50%	84.30%	89.97%	87.99%
	DoS GoldenEye	13260	90.56%	98.17%	98.53%	98.06%
	**Total**	**23454**	**85.97%**	**93.52%**	**95.64%**	**94.87%**
DARPA98	Smurf	5013	81.87%	90.01%	92.34%	92.16%
	IPsweep	11326	73.64%	78.15%	81.69%	80.53%
	Warez	1786	85.72%	88.43%	96.70%	95.30%
	Rootkit	254	79.53%	95.81%	94.49%	90.94
	**Total**	**18379**	**77.14%**	**84.30%**	**86.23%**	**85.34%**

**Table 5:** Comparison of the time complexity of different algorithms.

	No-Weight Method	Scheme 1 [29]	Scheme 2 [31]	Our Scheme
Attribute Weight Determination	O(1)	$O\left({mu}^{2}\right)$	$O\left({mu}^{2}\right)$	$O\left({mnu}^{2}\right)$
Alert Aggregation	$O\left(u^{2}\log u\right)$	$O\left(u^{3}\right)$	$O\left(u^{3}\log u\right)$	$O\left(u^{2}\log u\right)$
